# A novel egg-shell membrane based hybrid nanofibrous scaffold for cutaneous tissue engineering

**DOI:** 10.1186/s13036-019-0208-x

**Published:** 2019-10-26

**Authors:** Leila Mohammadzadeh, Reza Rahbarghazi, Roya Salehi, Mehrdad Mahkam

**Affiliations:** 1 Chemistry Department, Faculty of Science, Azarbaijan Shahid Madani University, Tabriz, Iran; 2 Stem Cell research Center, Tabriz University of Medical Sciences, Tabriz, Iran; 3 Drug Applied Research Center and Department of Medical Nanotechnology, Faculty of Advanced Medical Science, Tabriz University of Medical Science, Tabriz, Iran

**Keywords:** Nanofibrous scaffolds, Basal cells, Differentiation, Eggshell membranes, *Aloe vera*

## Abstract

**Background:**

The main issue in cutaneous regeneration is to develop engineered scaffolds based on natural extracellular matrix to promote dynamics of skin progenitor cells and accelerate differentiation into mature keratinocytes.

**Methods:**

In this study, nanofibrous scaffolds composed of a blend poly (ɛ-caprolactone) (PCL), silk fibroin (SF), soluble eggshell membrane (SESM), and *Aloe vera (*AV) gel were developed by electrospinning method and human basal cells were used to examine differentiation capacity toward keratinocyte-like cells. For this propose, cells were allocated to four distinct groups; control, PCL/SF, PCL/SF/SESM, and PCL/SF/SESM/AV. In all groups, cells were incubated with differentiation medium. Morphology, composition, hydrophilicity and mechanical features of PCL/SF, PCL/SF/SESM and PCL/SF/SESM/AV nanofibers were studied by scanning electron microscopy (SEM), Fourier transforms infrared spectroscopy (FT-IR), water contact angle and tensile tests. To examine the orientation of basal cells to mature keratinocytes, we performed immunofluorescence analysis by monitoring cytokeratin-19. The expression of genes such as involucrin, keratin-14 and -5 was monitored by real-time PCR assay.

**Results:**

PCL/SF, PCL/SF/SESM, and PCL/SF/SESM/AV had suitable physic chemical indices and biological activities to be applied as biomimetic scaffolds for the restoration cutaneous tissue. Compared to control, we found an increased basal cell proliferation at 7 and 14 days after plating on scaffolds and reach maximum levels in group PCL/SF/SESM/AV on day 14 (*p* < 0.05). Electron microscopy showed cell flattening, morphological adaptation. An integrated cell-to-cell connection was generated after cell seeding on scaffolds in all groups. Immunofluorescence imaging showed the ability of basal cells to synthesize cytokeratin-19 in PCL/SF, PCL/SF/SESM, and positive control cells after exposure to differentiation medium. However, these values were less in PCL/SF/SESM/AV compared to other groups. Real-time PCR analysis showed the potency of all scaffolds to induce the transcription of involucrin, keratin-14 and -5, especially involucrin in PCL/SF/SESM/AV group compared to the negative control.

**Conclusion:**

Modulation of scaffolds with natural biopolymers could enable us to synthesize structures appropriate for cutaneous regeneration.

## Introduction

Cutaneous tissue is the largest organ that protects the body against infectious agents, mechanical, chemical and thermal stresses while prohibits the evaporation of biofluids [1, 2]. In addition, immune surveillance, sensory detection, and self-healing correlate with the normal activity of the skin [3]. In most circumstances, this organ has the potential to heal without any external manipulations.

The existence of basal cells, namely basal cells, potentiates skin to replace injured cells with healthy keratinocytes. Basal cells are located at the interphase between epidermis and dermis layers. It is thought that basal cells proliferate in response to stimulatory clues and thereby transform to mature functional keratinocytes which are evident by the expression of specific factors such as cytokeratins, desmins, desmogleins, etc. [4]. Unfortunately, after the onset of extensive cutaneous diseases and wounds caused by erosion, burns, diabetic changes, and other traumatic events, this tissue is unable to promote reparative procedures and thereby therapeutic approaches are essential to circumventing these conditions [1, 5]. While autologous grafts are touted as the gold therapeutic approaches but these modalities are limited due to the lack of suitable donor, the possibility of immunologic responses [1, 5–8]. Therefore, de novo modalities, such as tissue engineering approaches, are needed to afford these limitations [9]. In this regard, the fundamental criteria of an appropriate scaffold are to maintain cell functional activity such as adhesion, proliferation, migration, differentiation, and morphology [7, 10].

From the past decades to the present time, among multiple techniques used to fabricate an efficient scaffold for cutaneous tissue engineering, electrospinning has been identified as an optimum technique in the preparation of scaffolds [11]. Considering the unidimensionality morphology to extracellular matrix (ECM) proteins, polymeric nanofibers fabricated via electrospinning has been found clinically appropriate biomaterials for skin repair [12]. Nanofibrous polymers could enhance the cell attachment, migration, and diffusion process because of the high surface/volume ratio. These features potentiate the cells to have active dynamic growth in nervous and cutaneous systems. Regarding the fact that most polymer scaffold properties such as hydrophilicity, biocompatibility and mechanical strength are associated with the type of polymer, thereby it is not possible to obtain all biological activities by using single composition. As a matter of fact, the combination of synthetic and natural polymers is thought to provide all above-mentioned features efficiently [13].

Natural eggshell membrane (ESM (is highly-collagenized fibrous tissue consists of typeΙ, V and X collagens [14–16]. In addition, the existence of essential structural proteins such as osteopontin, sialoprotein [17, 18], keratin, proteoglycans, and glycoproteins have been previously proved. Also, ESM harbors numerous natural glycoproteins notably glucosamine, chondroitin, and hyaluronic acid which are completely applicable for the cutaneous wound dressings [19, 20]. In spite of these advantages, disulfide bonds as cross-link, limit the solubility of ESM and its use in biomedical engineering, as the nanofibrous scaffold. However, Although Soluble ESM (SESM) is prepared by reduction dissolved method, due to its poor mechanical properties because of its low molecular weight and wide molecular weight dispersion, is make difficult to obtain a nanofibre or film form ESM [21]. Commensurate with these statements, the addition of an appropriate scaffold backbone seems essential to preserve this property. Poly(ɛ-Caprolactone) (PCL) possesses an excellent mechanical property [22]. Therefore, it seems that ESM with PCL mixture is the strategic approach to constitute nanofibrous scaffold with desirable mechanical and biocompatible properties [21].

Silk fibroin (SF) is produced by *Bombyx mori* silkworm and is fibrous proteins composed essentially of fibroin [23, 24]. Due to prominent biological properties, biocompatibility, minimal inflammatory reaction, biodegradability, and permeability silk-based nanofibers are at the center of attention for tissue engineering approaches [25–28]. The high strength of the nanofibrous scaffolds composed of silk is commonly applied for tissue engineering with the ability to maintain cell morphology, proliferation, and differentiation [29, 30].

*Aloe vera* (AV), as the oldest pharmaceutical herbs is a member of the Liliaceae family [31]. AV has the ability to be applied for the alleviation of multiple cutaneous pathologies peculiarly burns, infections, pain, and enhanced cell proliferation. The mucilaginous gel existed in AV leaf is composed of 99% water and long-chain polysaccharides mainly acetylated glucomannan and numerous carbohydrates. AV gel is integral to wound hydration due to higher water content (~ 99%) [32–40]. The existence of high osmotic value provided by glucose prohibits pathogenic bacteria. AV glycoprotein fraction was previously found to accelerate cell proliferation and migration of fibroblasts and keratinocytes [38].

In the current experiment, we aimed to investigate the regenerative potential of PCL/SF, PCL/SF/SESM, and PCL/SF/SESM/AV scaffold as natural biomaterials on the differentiation of human basal cells to keratinocytes over a period of 14 days.

## Materials and methods

### Materials

In this study, PCL (Mw = 80,000; Cat no; 24,980–41-4), NaHCO_3_, CaCl_2_, were purchased from Sigma-Aldrich (Co., Steinem, Germany). The 3-mercaptopropionic acid, acetic acid, sodium hydroxide (NaOH), CH_3_CH_2_OH, formic acid, and methanol were obtained from Merck Chemical Co. Specific-pathogen-free eggs were obtained from poultry husbandry (East Azerbaijan, Iran), *Bombyx mori* cocoons were purchased from Tabriz Traditional Carpet Market and fresh AV leaves were collected from plants (purchased from the Iranianbotanic shop). Phosphate-buffered saline (PBS) and fetal bovine serum (FBS), Dulbecco’s modified eagle medium (DMEM-F12), were obtained from Gibco. 3-(4, 5-dimethylthiazol-2-yl-2, 5-diphenyltetrazolium bromide) (MTT) was supplied from Invitrogen (Carlsbad, CA), DAPI (4,6-diamidino-2-phenylindole) (Cas no; 28,718–90-3), growth factors contain: EGF (cat no;213–10,068), KGF (cat no; 213–10,172) and IGF (cat no;213–10,172) and cytokeratin-19 (Cat no: ab178543; Abcam).

### Preparation of the soluble eggshell membrane

The Fresh eggshell membrane (ESM) was peeled and dissolved in the mixture containing 1.5 M of 3-mercaptopropionic acidand 10% acetic acidand kept at 90 °C for half of the day. After cooling to room temperature, insoluble components were excluded by centrifugation (at 15000 rpm for 15 min). The pH of the solution was set to 5 by using NaOH (5 M). After filtration of solutions, supernatants were discarded and precipitants wash with pure methanol and finally to obtainthesoluble eggshell membrane (SESM) was lyophilized.

### Preparation of regenerated silk fibroin (SF) solution

In the current experiment, cocoons of *Bombyx mori* silkworm silk were applied to fabricate SF nanofibers. First, the cocoons were chopped into small sizes and boiled twice in sodium carbonate solution (0.5 wt%) for 30 min to scour and clean the surface of cocoons. For sericin removal, cocoons were impregnated inside warm distilled water and dried overnight. Next, degummed SF was dissolved by using a ternary solvent system consisted of CaCl_2_/CH_3_CH_2_OH/H_2_O (with a molar ratio of 1: 2: 8, respectively) at 70 °C for 6 h. Afterward, the mixture was dialyzed via tubular cellulose membranes in distilled water over a period of three days. In order to obtain regenerated SF sponges, SF solution was finally freeze-dried.

### Preparation of eggshell, SF and PCL solutions

For electrospinning, we prepared working solutions by dissolving 13.5 wt% SF and SESM individually in formic acid and PCL was dissolved with final concentrations of 10 wt% in the acetic acid/formic acid (30/70) solvent mixture. The solutions were gently stirred at RT for three hours until a homogenous solution appeared. Finally, SF and PCL solutions were mixed with volume ratio 15:85 and SF, SESM and PCL solutions prepared with a volume ratio of 15:15:70. To synthesize AV nanofibers, 15% (w/w) AV, calculated based on the total weight of applied polymers in the final solution, was mixed with PCL/SF/SESM solution and stirred for next 1 h. All solutions were vigorously mixed at ambient temperature for 12 h followed by placing in a 5 ml plastic syringe which connected to a 22-gauge blunt needle. Electrospinning procedure was carried out at RT (22 ± 2 °C) under a humidified atmosphere (65 ± 5%). The electrospinning procedure was done by a high-voltage source (17 kV) and needle tip placed at a distance of 10 cm from the collector. Polymeric solution flow rate was adjusted to 0.5 ml per hour. The prepared mats were then completely dried under vacuum condition for 24 h to exclude any residual solvent.

### Characterization

The characteristic of nanofibrous scaffolds was monitored *by* scanning electron microscopy (SEM) (Prox, Phenom CO, Netherlands) after sputter-coating with gold. The diameters of the nanofibers were measuredby analyzing SEM images using appropriate software (Image J; NIH). The chemical constituents of the nanofibers were confirmed using FT-IR spectroscopy (Bruker, Tensor 27 spectrophotometer, Germany) in the wave number range of 400–4000 per centimeter. The surface wettability of each sample was evaluated by water contact angle analyzer (Data Physics Instruments GmbH, Germany). A droplet of deionized water with a size of 5 s was dropped onto the surface of the nanofibrous scaffolds and photographs were taken at 5 s to evaluate the hydrophilic nature of nanofibers. The tensile properties of the electrospun nanofibers were performed using a Zwick tensile tester (Z010, Zwick/Roell, Ulm, Germany). Rectangular samples of PCL/SF, PCL/SF/SESM, and PCL/SF/SESM/AV nanofibers with a width of 10 mm and length of 50 mm were utilized for testing with a 10 N low-force load cell and at a crosshead speed of 5 mm/min. Tensile stress, strain as well as elastic modulus were calculated based on the resulted tensile stress-strain curves [41].

### In vitro degradation

In-vitro degradation test was done based on the method reported previously [42]. All samples cut in to small (10 × 10 mm) pieces. First dry nanofibers weighed (W_0_) and then immersed in PBS (pH = 7.4; Sigma) and were placed in incubator in 37 °C for a special period of time. After each time interval, the nanofibers were taken out from the PBS, gently washed with ultrapure water, dried at room temperature, and then weighed (W_t_). Values are mean ± SD (*n* = 3). The polymer weight loss (%) of samples was computed from the following formula:

$$ \mathrm{Weight}\ \mathrm{loss}\ \left(\%\right)=\left({\mathrm{W}}_0\hbox{-} {\mathrm{W}}_{\mathrm{t}}\right)/{\mathrm{W}}_0\times 100 $$

### Cutaneous basal cell expansion

In the current experiment, human cutaneous basal cells were used to investigate whether scaffolds have the potential to promote cell differentiation toward mature keratinocytes. For this proposes, human basal cells were obtained from the Stem Cell Research Center Cell Baking (Tabriz University of Medical Sciences) that obtained previously from human skin samples [4]. To expand the BCCs, we used epithelial cell growth basal medium (Cat no: CC-3151; Lonza) containing growth factor cocktail such as keratinocyte growth factor, insulin-like growth factor, heparin, hydrocortisone, fibroblast growth factor, epidermal growth factor, ascorbic factor (CC-4136; Lonza), 1% Pen-Strep (Gibco) and 10% fetal bovine serum (Gibco). Cells were detached at 70–80% confluence by using an enzymatic solution Trypsin-EDTA (0.25%; Gibco). Cells at passages 3–5 were used in the various analyses. The exhausted medium was replenished every 3–4 days.

### Characterization of isolated basal cells by flow cytometry

To characterize the isolated basal cells, flow cytometry analysis was performed. In brief, cells at passage 3 were collected, blocked withFBS and permeabilized by permeabilizing buffer (eBioscience) for 20 min. Thereafter, cells were incubated with anti-human CD10 antibody (FITC-conjugated; F0826; Dako) for 30 min. After three wash with phosphate-buffered saline (PBS), the percent of CD10 positive BCC was calculated by using BD FACSCalibur apparatus and FlowJo software (version 7.6.1.).

### Cell seeding on different nanofibrous scaffolds and MTT assay

Prior to cell seeding, nanofibers were disinfected with 70% EtOH, then dried in the air and placed under UV light for 20 min on each side. Thereafter, the scaffolds were washed with PBS and kept in culture medium for a period of 24 h in order to facilitate an optimal cell adhesion rate.

An initial number of 1 × 10^4^ cells was re-suspended in 200 μl DMEM/F-12 medium with 2% FBS and transferred to per well of 96-well plates (SPL) pre-coated withPCL/SF, PCL/SF/SESM, and PCL/SF/SESM/AV scaffolds. At respective time points 7 and 14 days, the medium was discarded and 200 μl MTT solution (3-(4, 5-dimethylthiazol-2-yl)-2, 5-diphenyltetrazolium bromide; 2 mg/ml) poured into wells and maintained at 37 °C in a 5% CO_2_ incubator for 4 h. Next, MTT solution was carefully pipetted out from the wells and dimethyl sulfoxide (DMSO; 150 μl/per well) used to dissolve formazan crystals. The absorbance of each well was recorded at 570 nm by amicroplate reader (Multiskan MK3, Thermo Electron Corporation, USA). This assay was repeated in sextuplicate.

### Scanning microscopy (SEM)

After 7 and 14 days, the morphological feature of BCC was examined by SEM analysis cultured on the surface of nanofibrousscaffolds. At both time points, the cells mass was washed with PBS (twice each for 5 min) to exclude non-adherent cells followed by incubation in 4% glutaraldehyde solution for 2 h followed by the incubation in increasing gradient of ethanol (50, 75, 90, and 100%). Then,samples were air-dried and coated with gold, and cell morphologies were evaluated by SEM.

### Immunofluorescence imaging (IF)

BCCs were culture on PCL/SF, PCL/SF/SESM, and PCL/SF/SESM/AV nanofibrous scaffolds with epithelial cell growth basal medium containing growth factor cocktail. BCCs cultured on tissue culture plate with and without growth factor were considered as positive and negative control. 14 days after cell culture on various scaffolds, cells were washed twice with PBS and fixed by using pre-chilled paraformaldehyde solution (4%) for 20 min. Nanofibrous scaffolds with cells were snapped frozen in optimal cutting temperature compound (OCT; Cat no: 4583). Cryostat-sectioned at the 5-μm thickness (Leica) were prepared. The slides were washed with PBS and permeabilized and incubated with 1% FBS for 20–30 min. Thereafter, mouse anti-human cytokeratin − 19 (dilution: 1:100; Abcam) was used to confirm the differentiation of BCC to keratinocyte-like cells. For this proposes, slides were maintained at room temperature for 1 h and washed with PBS (three times each for 5–10 min). For nuclear staining, we used DAPI solution. Slides were visualized by fluorescent microscopy (Model: BX51; Olympus) and imaged processed by Cell Sense software.

### Real-time PCR analysis

To confirm the differentiation of BCCs to keratinocyte-like cells, the expression of three relevant genes such as IVL, KRT-14, and KRT-5 was studied by real-time PCR analysis. BCCs were culture on PCL/SF, PCL/SF/SESM, and PCL/SF/SESM/AV nanofibrous scaffolds with epithelial cell growth basal medium containing growth factor cocktail. BCCs cultured on tissue culture plate with and without growth factor were considered as positive and negative control. Fourteen days after cell culture samples were collected and rinsed with PBS. Thereafter, total RNA was extracted using Tri-Reagent (Sigma, USA). Thereafter, total RNA Kit (Bio basic, Toronto, Canada) was utilized to extract the total RNAs from all of the treatment samples and hDPSCs used as control group [43]. The gel electrophoresis and Nanodrop (Thermo Scientific, Waltham, MA, USA) were applied for the determination of extracted RNA yield and value. Isolated total RNAs (1 μg) was utilized for cDNA synthesis using a Revertide cDNA synthesis kit (Fermentase, Life Science, USA). Isolated RNAs (1 μg) was reverse-transcribed using to cDNAs. Each reaction mix consisted of 0.5 μg of hexamer random primer/1 μg of the total RNA. The mixture was heated (65 °C for 5–10 min) and instantly cooled on ice. In this study, 20-μL reaction volume included 0.5 mM dNTP, 25 IU (unit) RNase inhibitor and 200 IU reverse transcriptase. This mixture was heated at 42 °C for 60 min. Strand synthesis mixture was done by SYBR Green PCR Master Mix and BioRad system. Real-time PCR reaction (20 μL) was done with a solution containing 200 nM primers and 10 μL SYBR Green Master Mix. This assay was done in triplicate (Table 1).

**Table 1:** Sequences of primers used for QRT-PCR

Gene	Sequence	
	Forward	Reverse
IVL	CCTTACTGTGAGTCTGGTTGAC	TGTTTCATTTGCTCCTGATG
KRT-14	CCGCACCAAGTATGAGACAG	TCAGGCTCTCAATCTGCATC
KRT-5	CCGTGCCGCAGTTCTATATT	ACTTTGGGTTCTCGTGTCAG
GAPDH	GGTGTGAACCATGAGAAGTATGA	GAGTCCTTCCACGATACCAAAG

### Statistical analysis

Data are shown in mean ± SD. We used One-Way ANOVA with Turkey analysis to find statistical differences between groups. *P-*values of less than 0.05 were considered statistically significant. All experiments were performed in triplicate otherwise mentioned.

## Results and discussion

### Morphology of electrospun nanofibers

**Fig. 1:**
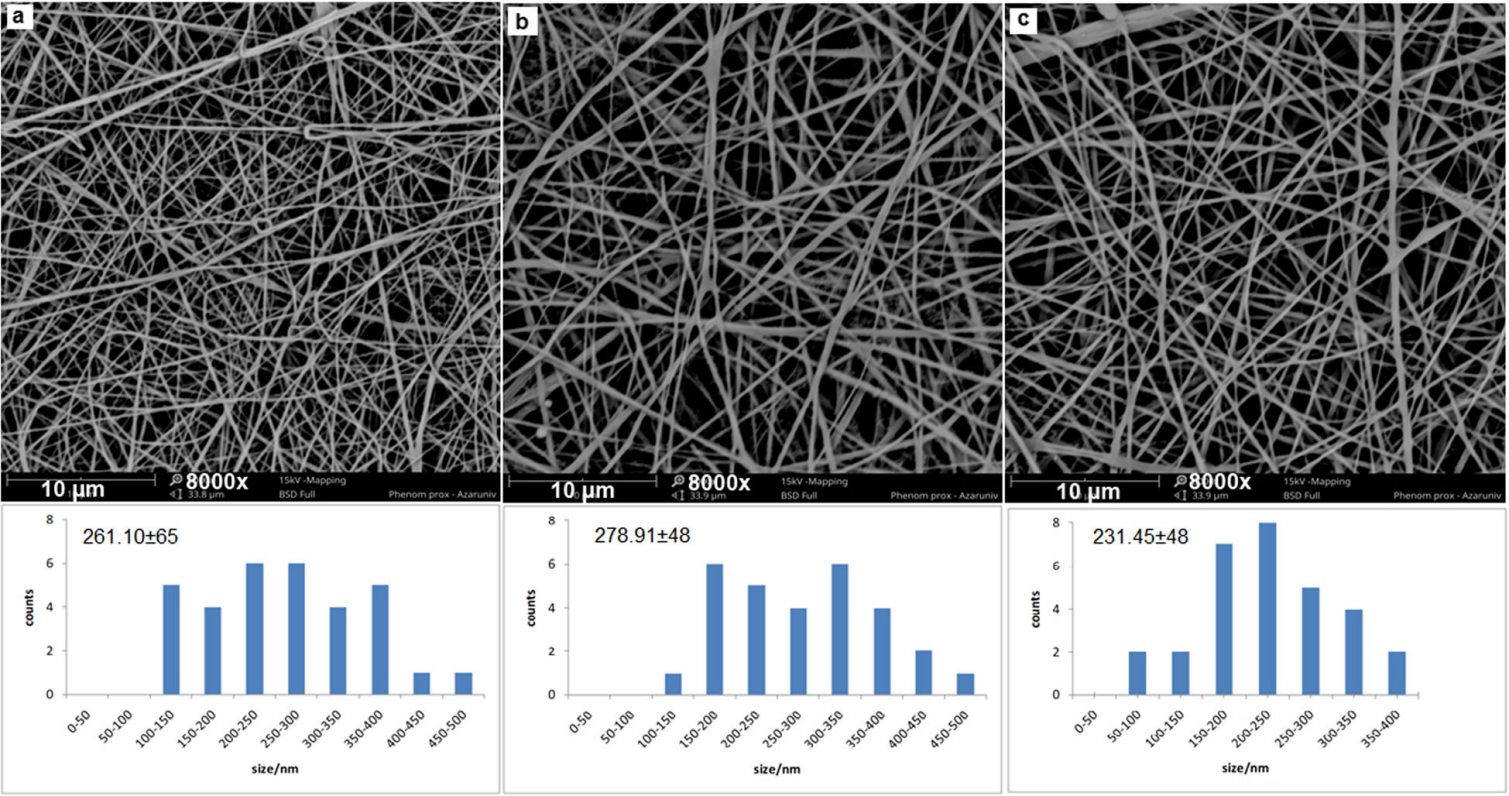
SEM micrographs of (**a**) PCL/SF, (**b**)PCL/SF/SESM, (**c**)PCL/SF/SESM/AV, nanofibers and the corresponding diameter distributions. Scale bar = 10 μm

The most interesting techniques for providing excellent nanostructured material for tissue engineering applications are electrospinning of polymer blends. Preparation of electrospun polymer blend nanofibers using trifluoroethanol (TFE), and 1, 1, 1, 3, 3, 3-hexafluoro-2-propanol (HFIP) [44–47] were carried out before. Because of the toxicity and high cost, the application of these solvents was limited, Only the acetic acid/formic acid solvent system seems applicable in PCL/SF, PCL/SF/SESM and PCL/SF/SESM/AV electrospinning due to the superior conductivity of formic acid which makes possible to the production of high-quality nanofibers [43]. With optimization of electrospinning parameters such as applied voltage, flow rate, tip-to-collection plate distance, concentration of the polymer solution, and the ratio of ingredients in the blend, beadles, randomly arranged, uniform nanofibers of PCL/SF, PCL/SF/SESM and PCL/SF/SESM/AV were prepared as shown in Fig. 1. SEM images of nanofibers showed PCL/SF and PCL/SF/SESM nanofibers exhibited a circular cross-section with a smooth surface. A small increase in nanofibers diameter was shown by the addition of SESM in the PCL/SF nanofibers; possibly because of the increase in viscosity of electrospinning polymer solutions. Figure 1 showed the diameter distribution of nanofibrous scaffolds. It appears that the addition of *Aloe vera* gel resulted in the fabrication of thinner nanofibers, because of the decreasing of the viscosity of the prepared solution for nanofiber production. Suganya et al. had previously reported the addition of AV to the PCL solution had a similar effect on the mean diameter of PCL nanofibers [33, 38]. Like other reports, it is obvious that the incorporation of AV mainly reduced the nanofiber mean diameter as a result of increased solution conductivity and decrease in solution viscosity [35].

### FT-IR analysis

**Fig. 2:**
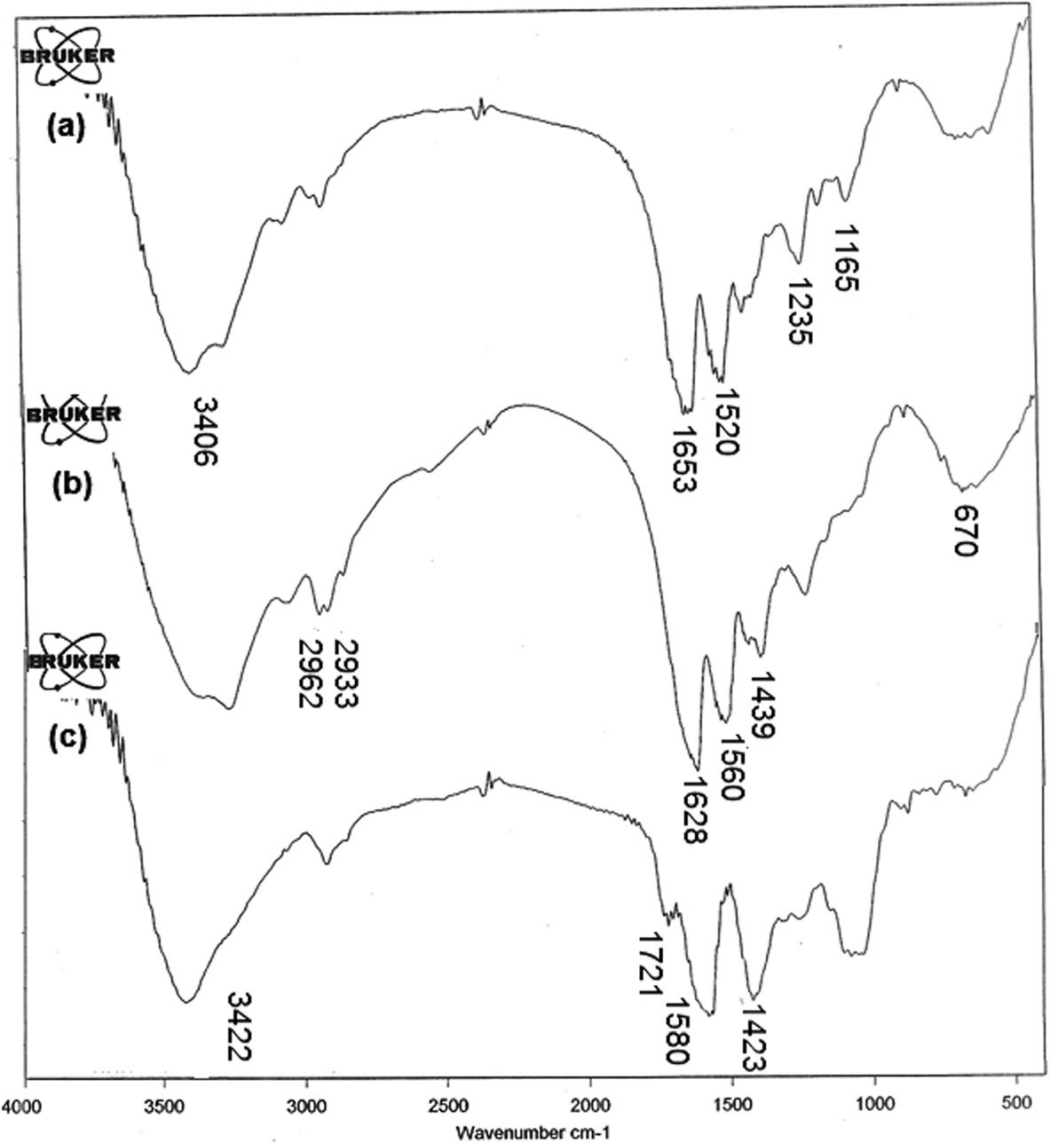
FT-IR spectra of (**a**) SF, (**b**) SESM, and (**c**) AV.

**Fig. 3:**
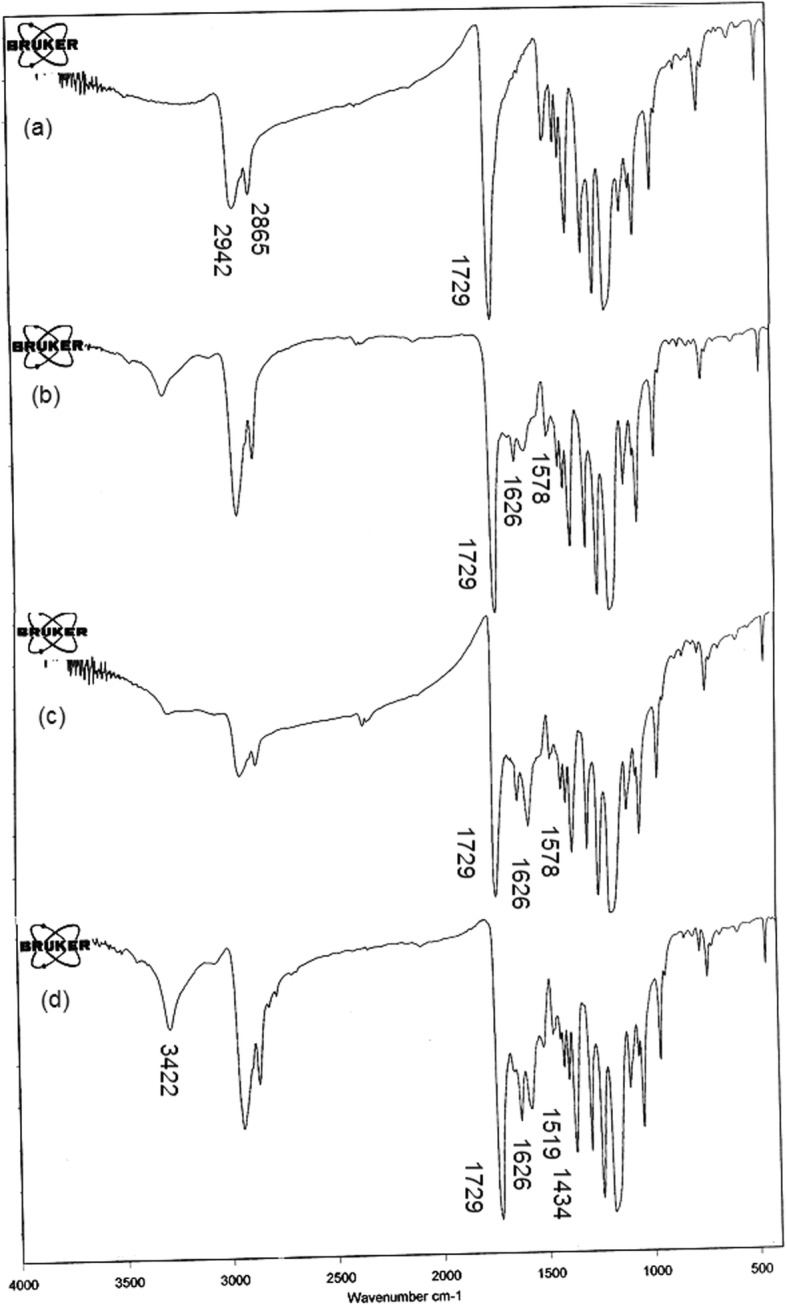
FT-IR spectra of (**a**) PCL, (**b**)PCL/SF, (**c**)PCL/SF/SESM and (**d**) PCL/SF/SESM/AV nanofibers

The characteristic features of SF, SESM and AV are shown in the spectra Fig. 2. The SF spectrum in Fig. 2(a) indicates respective peaks at, 1653 cm^− 1^ amide-I, 1520 cm^− 1^ for amide-II, 1165 and 1233 cm^− 1^ for amide-III, and at 3406 cm^− 1^ which was related to N-H stretching vibration (secondary amide) hydroxyl groups presents in SF structure [48] and the SESM spectrum is display the most intensive band at 3286 cm^− 1^ is related to the stretching vibrations of O-H and N-H groups. Peaks at 2962 and 2933 cm^− 1^ correspond to the asymmetric stretching vibrations of the = CH_2_ and symmetric = CH_2_ stretching, respectively. The bands at 1628 cm^− 1^ (amide C=O stretching), 1560 cm^− 1^ (amide N–H bending), 1439 cm^− 1^ (CH_2_ scissoring), and 670 cm^− 1^ (C–S) were observed [14]. The spectrum of AV exhibits an absorption peak at 3422 cm^− 1^, which related to O–H vibration of phenolic groups in the AV [49]. The peak at 2920 cm^− 1^ is corresponds to the symmetrical and asymmetrical (C–H) stretching the CH_2_, which are also characteristic of the existence of aliphatic (C–H) groups in the structure of AV. The peak at 1721 cm^− 1^ is corresponds to (C=O (stretching mod and show the presence of carbonyl groups. The intense band at 1580 cm^− 1^ is related to)C=C(stretching, which represent the presence of vinyl ether and aloin compound [50]. absorption bands are appeared in the range of 1400–1600 cm^− 1^, are attributed to the aromatic ring (C=C) [49]. The FT-IR spectra of PCL, PCL/SF, PCL/SF/SESM, and PCL/SF/SESM/AV nanofibers are shown in Fig. 3. PCL spectrum shows the absorption band of the carbonyl group (C=O) at 1729 cm^− 1^, CH_2_ stretching vibrations at 2942 cm^− 1^ (asymmetric) and 2865 cm^− 1^ (symmetric) [29], 1294 cm^− 1^ (C-O and C-C stretching) [51], 1241 cm^− 1^ (asymmetric C-O-C stretching), C–O–C stretching at 1181 cm^− 1^ (symmetric). The spectral result for PCL/SF nanofiber all of the characteristic peaks of PCL and new additional peaks appeared at the region of 1626 and 1578 cm^− 1^ related to the amide-I and -II bands in the SF. FT-IR results confirmed the existence of SF in the nanofiber. After incorporating SESM in PCL/SF mats, the characteristic peaks of SESM in addition to peaks of PCL/SF appeared. Unfortunately, the majority of the characteristic peaks of SESM lied in an area overlapped by PCL/SF absorption peaks except for an increase in the intensity of peaks at the region of 1626 and 1578 cm^− 1^ which related to the amide -I and -II bands in the SESM. The presence of AV was confirmed by the peaks at 1516 and 1434 cm^− 1^ correspond to stretching vibrations of aromatic (C=C), also increasing the O–H vibration of phenolic groups in the structure of AV confirmed the presence of AV in PCL/SF/SESM/AV.

**Fig. 4:**
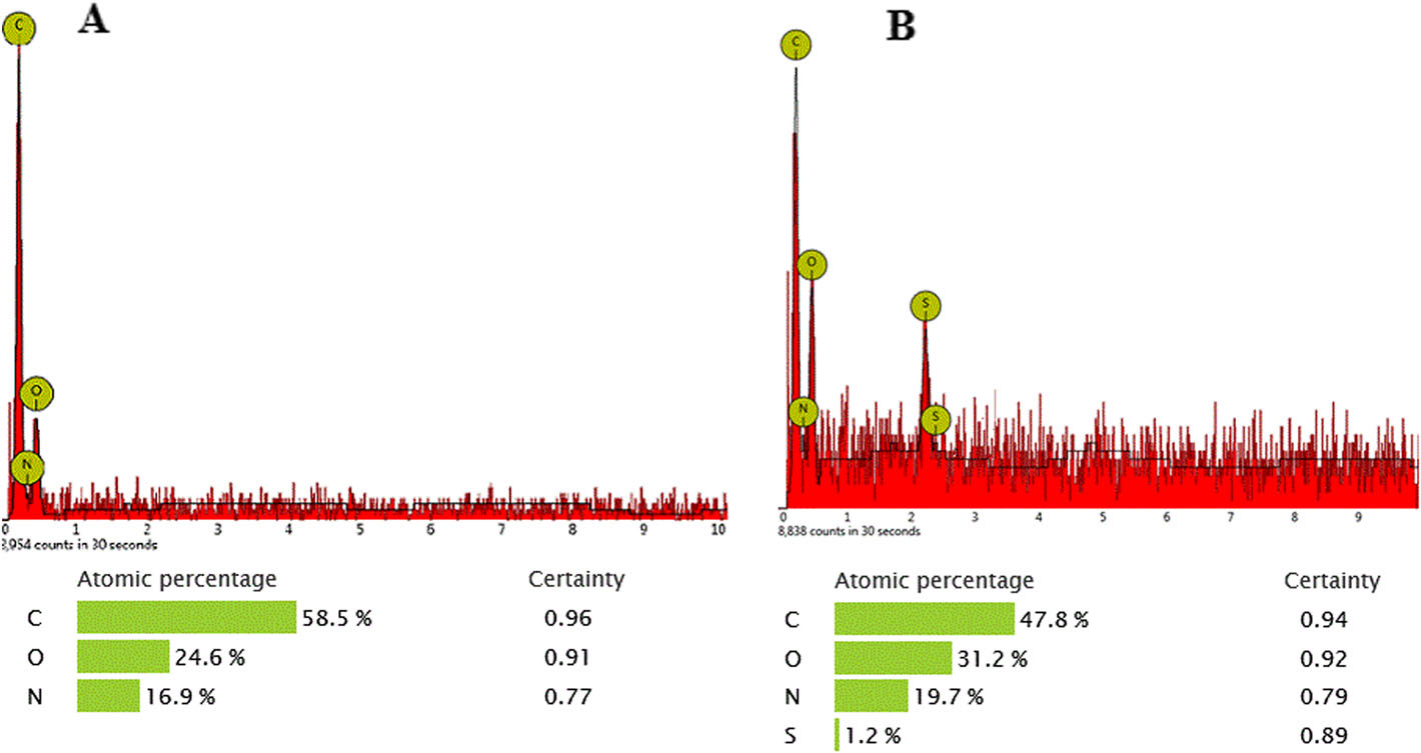
Corresponding EDX pattern of (**a**) PCL/SF, (**b**) PCL/SF/SESM nanofibers

Moreover, the elemental composition of the as-prepared PCL/SF and PCL/SF/SESM composite materials was also examined by EDX. Results in Fig. 4 showed S element in the SESM group due to the presence of sulfur-containing groups. The S peak was absence in the spectrums of PCL/SF nanofibers, which provides a piece of supportive evidence for the existence of SESM on the PCL/SF/SESM nanofibrous scaffolds [52].

### Water contact angle analysis

**Fig. 5:**
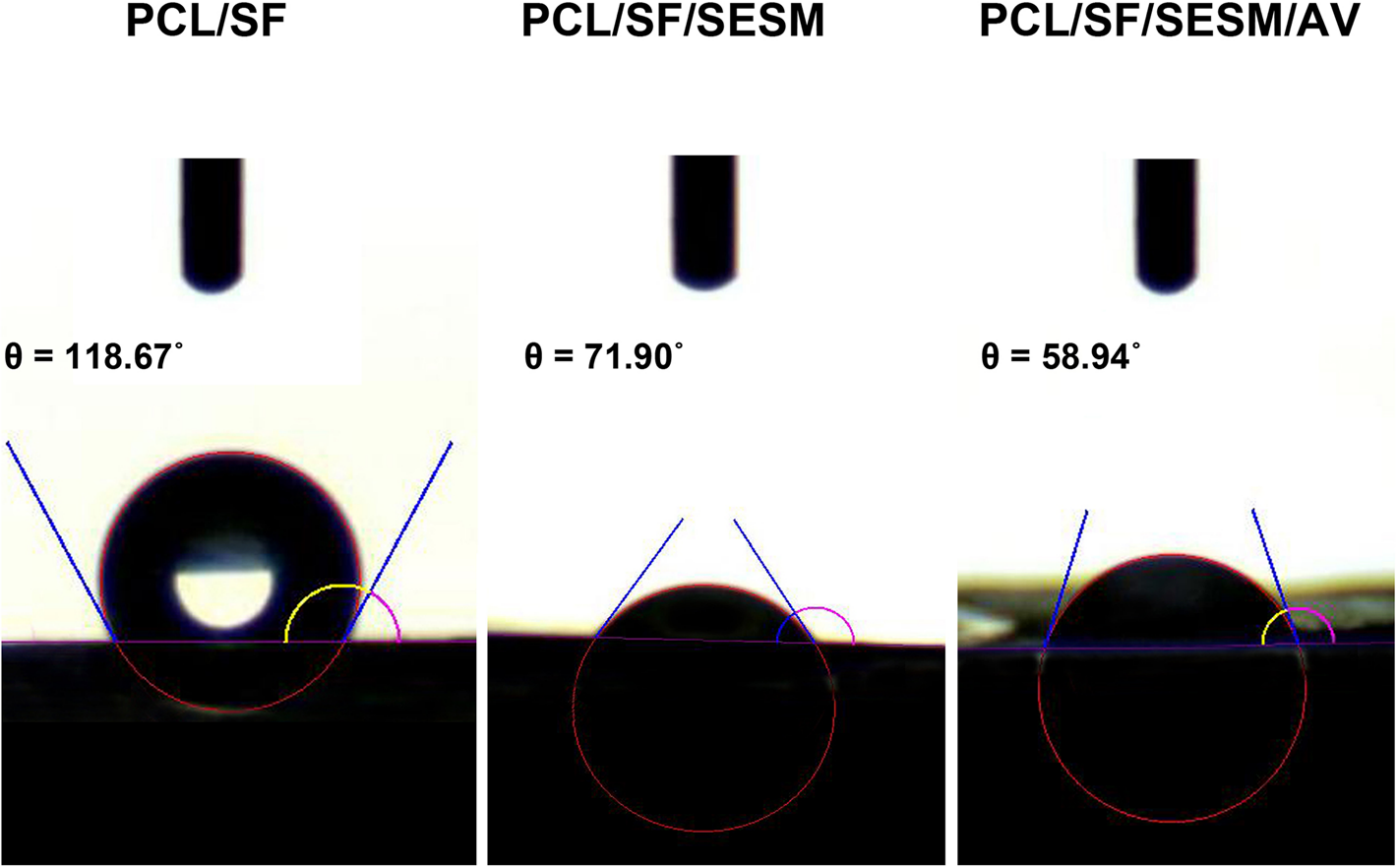
Water contact angles of electrospun scaffolds: PCL/SF, PCL/SF/SESM and PCL/SF/SESM/AV

The surface hydrophilicity of biomaterials is one of the important characteristics since it can influence initial adhesion, migration, proliferation and viability of cells [53]. Considering the significance of surface hydrophilicity of the biomaterial for biomedical applications, specifically tissue engineering, hydrophilicity of the PCL/SF, PCL/SF/SESM and PCL/SF/SESM/AV nanofibrous scaffolds was determined. The results of water contact angle (WCA) were displayed in Fig. 5, which indicate a reduction in WCA index of PCL/SF with the incorporation of SESM. By increasing SESM component in nanofibrous blend, the hydrophilicity was enhanced which probably attributed to the higher solubility of SESM by reduction of disulfide bonds [20]. The presence of SESM contents in the PCL/SF/SESM nanofibers increases water absorbency of the nanofibrous scaffolds which is shown by the decrease of WCAs from 118.67° ± 1.71° to 71.90° ± 1.08°in PCL/SF nanofiber webs. The incorporation of AV in PCL/SF/SESM/AV nanofibrous scaffolds altered the surface features of nanofibrous scaffolds due to the soluble and nature of AV [35]. As shown in Fig. 5, the presence of AV, decrease the WCA from 71.90 °± 1.08°to 58.94 ° ± 2.1°. The presence of large amount of polysaccharides, protein, and other hydrophilic special compounds in AV structure could expose hydrophilic groups on the scaffolds and increase hydrophilicity [35] (Table 2).

**Table 2:** Water contact angles of nanofibers

Scaffold	Contact angle
PCL/SF	118.67° ± 1.71°
PCL/SF/SESM	71.90° ±1.08°
PCL/SF/SESM/AV	58.94° ±2.1°

### Mechanical test

**Fig. 6:**
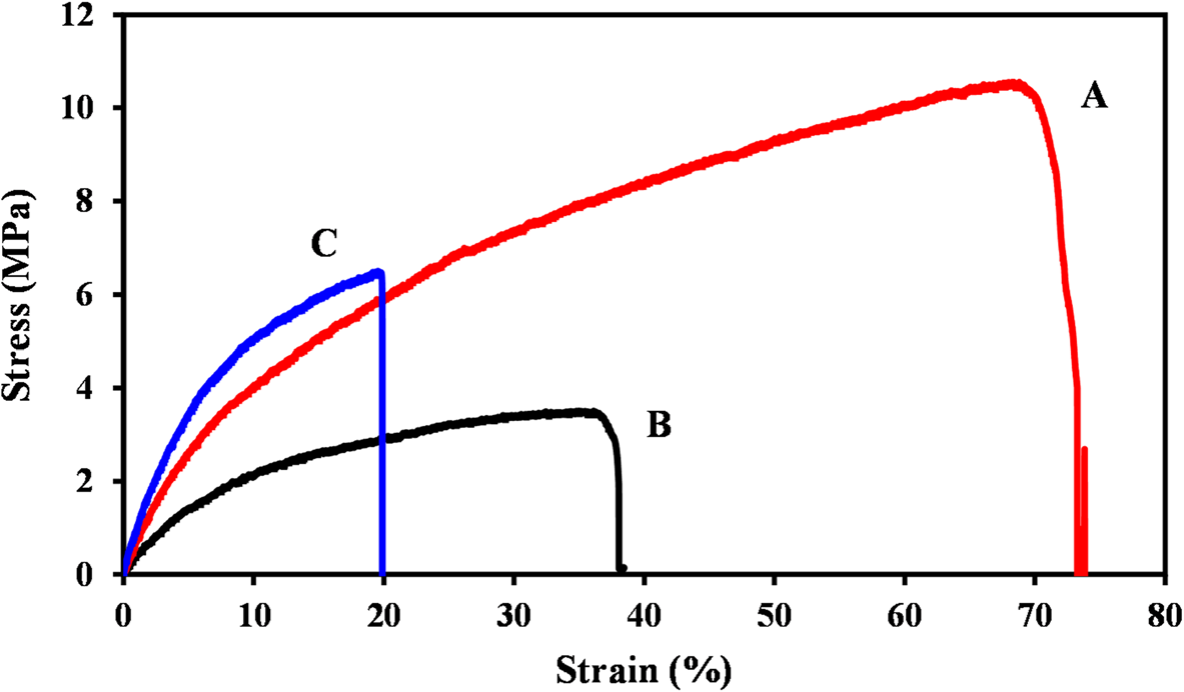
Stress–strain curve of (**a**) PCL/SF, (**b**)PCL/SF/SESM, (**c**) PCL/SF/SESM/AV nanofibers

The mechanical characteristics of the fabricated nanofibrous scaffolds are essential factors in preparing mechanical support for cellular adhesion and proliferation [54]. But the mechanical properties of nanofiber webs are poor, therefore, it is difficult to expose cultured cells to the correct stress environment in order to produce new tissues. To enhance the deficiency of mechanical properties of electrospun fibers, numerous studies have researched several blending systems, controlling processing parameters and post-processing treatments. The mechanical properties of nanofibrous scaffolds namely, PCL/SF, PCL/SF/SESM, PCL/SF/SESM/AV were evaluated (Fig. 6). Tensile strength for PCL/SF, PCL/SF/SESM and PCL/SF/SESM/AV nanofiber webs was 10.52 MPa, 3.46 MPa and 6.40 MPa can withstand a strain of 73.81, 38.04, and 19.87%, respectively. The results revealed that the incorporation of SF provided excellent mechanical behavior [48] in PCL/SF group. After the addition SESM, the tensile strength and elongation at break were reduced since of the intrinsic weakness of SESM compared with electrospun PCL/SF [46]. By adding AV, tensile strength increased for PCL/SF/SESM/AV fibers. The mechanical strength in PCL/SF/SESM/AV in comparison with PCL/SF/SESM nanofibers was enhanced due to polysaccharide richness in AV gel [35]. Despite PCL/SF/SESM nanofibers presenting low tensile strengths compared with PCL/SF and PCL/SF/SESM/AV scaffolds, the produced nanofibers show favorable mechanical characteristic which is sufficient for satisfying wound healing (Table 3).

**Table 3:** Mechanical properties of nanofibers

Sample	Tensile strength (MPa)	Elongation at break (%)	Young’s modulus (MPa)
PCL/SF	10.52 ± 0.55	73.81 ± 0.36	75.29 ± 0.25
PCL/SF/SESM	3.46 ± 0.63	38.04 ± 1.2	36.14 ± 1.2
PCL/SF/SESM/AV	6.40 ± 2.09	19.87 ± 5.2	125.03 ± 0.89

### In vitro degradation

**Fig. 7:**
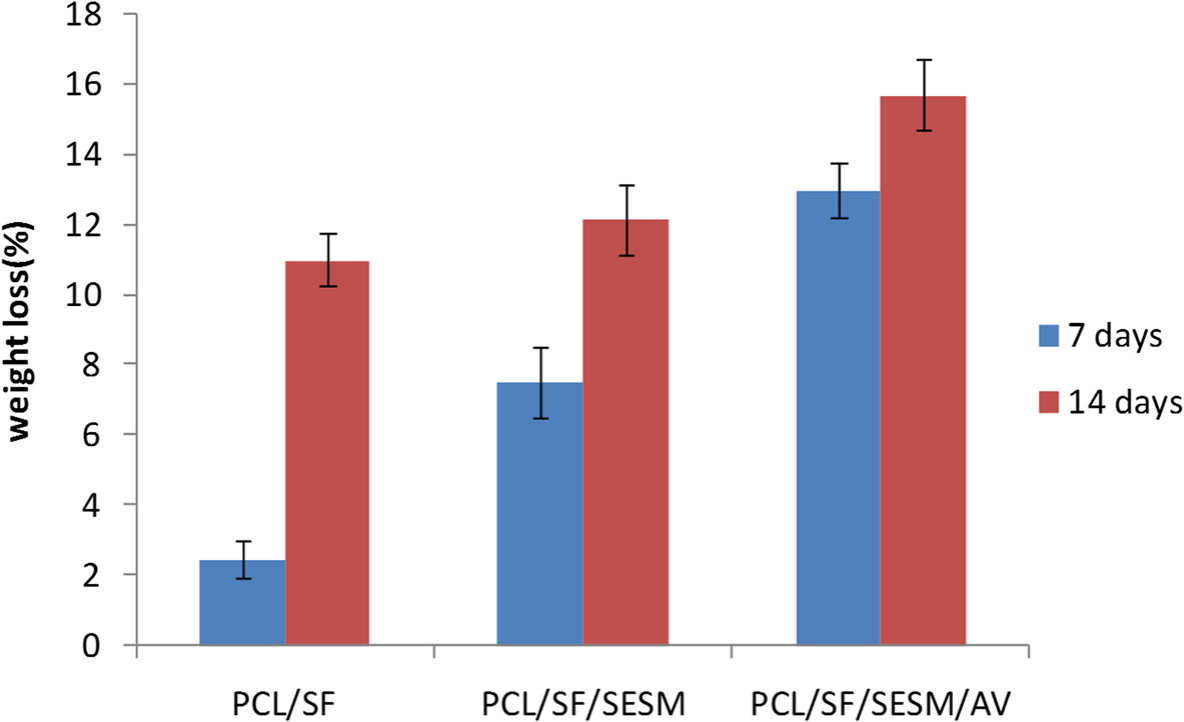
In-vitro biodegradation test of PCL/SF, PCL/SF/SESM, PCL/SF/SESM/AV nanofibers after 7 and 14 days of incubation

Fig. 7 indicated weight loss of PCL/SF, PCL/SF/SESM and PCL/SF/SESM/AV nanofibers after 14 days of incubation in PBS solution in 37 °C. As Fig. 7 exhibits the rate of degradation improved with increase of degradation time, at the first week the weight loss was minor in all nanofibers. The degradation rate was further increased after 14 days incubation in PBS. Obviously the mass decrease observed for PCL/biopolymer nanofibers comes from existence biopolymers (SF, SESM and AV) leaching only. According to results, the degradation rate of nanofibers improved with the increase of biopolymers content. These results might be due to high hydrophilicity of nanofibrous scaffolds as explained in water contact angle analysis [42].

### BCC morphology and characterization

**Fig. 8:**
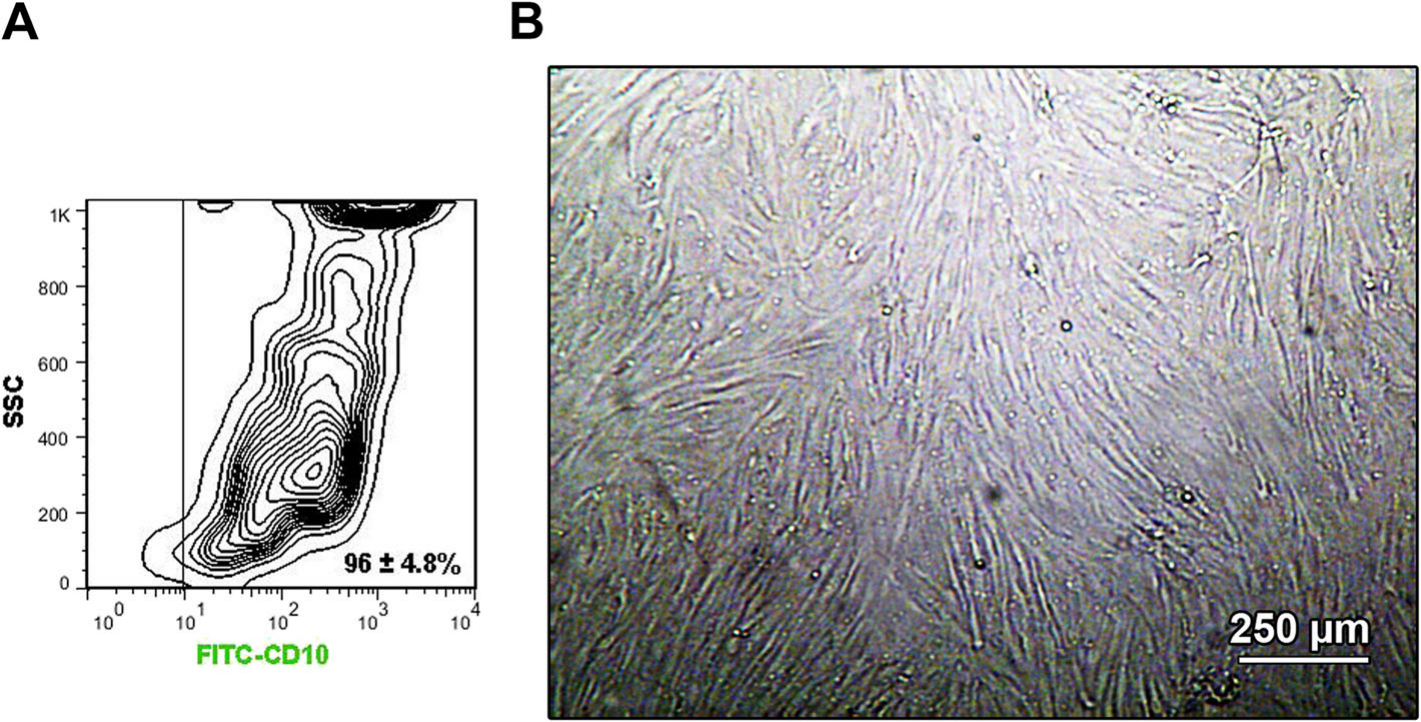
(**a**)Flow cytometry analysis of BCCs based on CD10 (FITC-tagged) at passage three. Data showed the existence of CD10 positive BCCs reaching 96 ± 4.8%, confirming a large number of BCCs in the current experiment (*n* = 3). (**b**) Bright field micrograph of BCCs at passage three. These cells showed fibroblast-like appearance and whirling pattern. This study was performed triplicate (*n* = 3)

Based on the bright field microscopic imaging, we noted that the isolated cells were spindle-shaped Fig. 8b. Flow cytometry analysis revealed the potency of isolated cells to express CD10, a BBC specific surface marker. In this panel, we found that over 90% of expanded BCCs were positive against the CD10, showing a high rate of cell homogeneity at passage three (Fig. 8a).

### Effect of PCL/SF, PCL/SF/SESM, and PCL/SF/SESM/AV nanofibers on BCCs viability

**Fig. 9:**
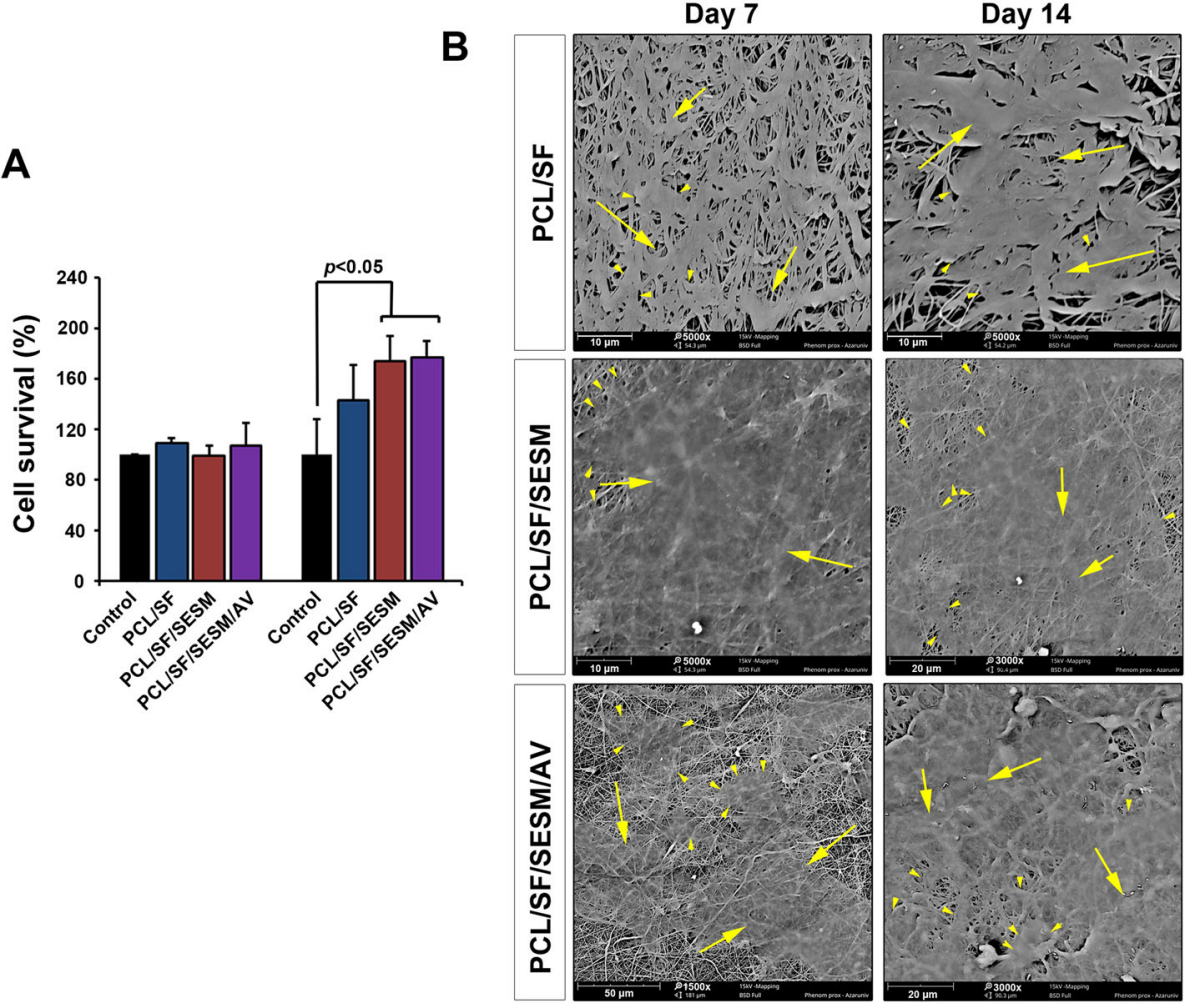
(**a**) Survival analysis of cells (MTT assay) on days 7 and 14. Data showed a significant increase in viability after plating on PCL/SF/SESM and PCL/SF/SESM/AV compared to matched-control groups at day 7 (***p < 0.001). This study was performed sextuplicate (*n* = 6). (**b**) SEM imaging of BCCs on the different nanofibers on days 7 and 14. It is shown that BCCs could easily attach the nanofiber surface and determined by a flattened appearance. In scaffolds such as PCL/SF/SESM and PCL/SF/SESM/AV cells tended to tightly connect with each other 14 days after plating. These features showed the ability of cultured cells to generate a uniform cell monolayer. This study was performed triplicate (*n* = 3). Arrow heads: cell margin; arrows cell mass

To evaluate the viability of BCCs after seeding on nanofibrous scaffolds, an MTT assay was carried out (Fig. 9a). In this regard, we measured survival values on days 7 and 14. MTT analysis showed that the cell viability was increased and reached significant levels 14 days after being cultured on PCL/SF, PCL/SF/SESM and PCL/SF/SESM/AV nanofibers while we found no statistically significant differences on survival rate on day 7. The addition of SESM and AV to PCL/SF backbone scaffold yielded higher cell viability compared to PCL/SF group (Fig. 9a). In support of this result, one reason would be related to the increase of cell attachment rate and scaffolds hydrophilicity due to a decrease of contact angle after the supplementation of PCL/SF with SESM and AV. Based on the data from WCA panel, PCL/SF/SESM nanofibrous scaffolds had a lower WCA index compared to PCL/SF nanofibrous scaffolds, contributing to the modulation of cell attachment. In addition, the mixture of hydrophilic agent SESM with PCL had the potential to provide numerous motifs and thereby increase an appropriate cell attachment area. Commensurate with these descriptions, the use of proteins and peptides in the synthetic scaffolds and composites promotes cell survival [54]. In addition to the advantages of proteinous substrates in nanofibers, it seems that the mixture of glycoprotein with backbone scaffolds could prominently increase the viability of the target cells. For instance, Choi et al. reported that by increasing the AV from 5 to 10% in the PCL structure mouse fibroblasts proliferation and migration was increased [38]. These results were inconsistent with our results obtained in this study. These features stand for a fact that the existence of numerous glycoproteins in scaffolds could increase cell proliferation and survival.

### SEM imaging showed BCCs attachment on nanofibers

SEM images of cultured BCCs on the PCL/SF, PCL/SF/SESM and PCL/SF/SESM/AV nanofibrous scaffolds showed cell attachment and flattening on day 7 (Fig. 9b). BCCs lost spindle-like shape after plating on nanofibers, showing morphological adaptation (Additional file 1: Figure S1). As shown in the SEM micrographs, BCCs showed a favorable attachment to the webs and efficiently distributed on the surface of the nanofibers. At day 14, large areas of scaffolds were covered by flattened BCCs and these cells tended to connect cell-to-cell juxtaposition by time. In ultramicroscopic evaluation, we found that PCL/SF/SESM and PCL/SF/SES/AV surfaces were efficiently covered by BCCs compared to PCL/SF after 14 days. These features demonstrated that the application of AV and SESM in the PCL-based scaffolds could force the BCC to form a unique monolayer which is required for epithelialization and cutaneous regeneration.

### IF staining revealed differentiation of BCCs to keratinocytes

**Fig. 10:**
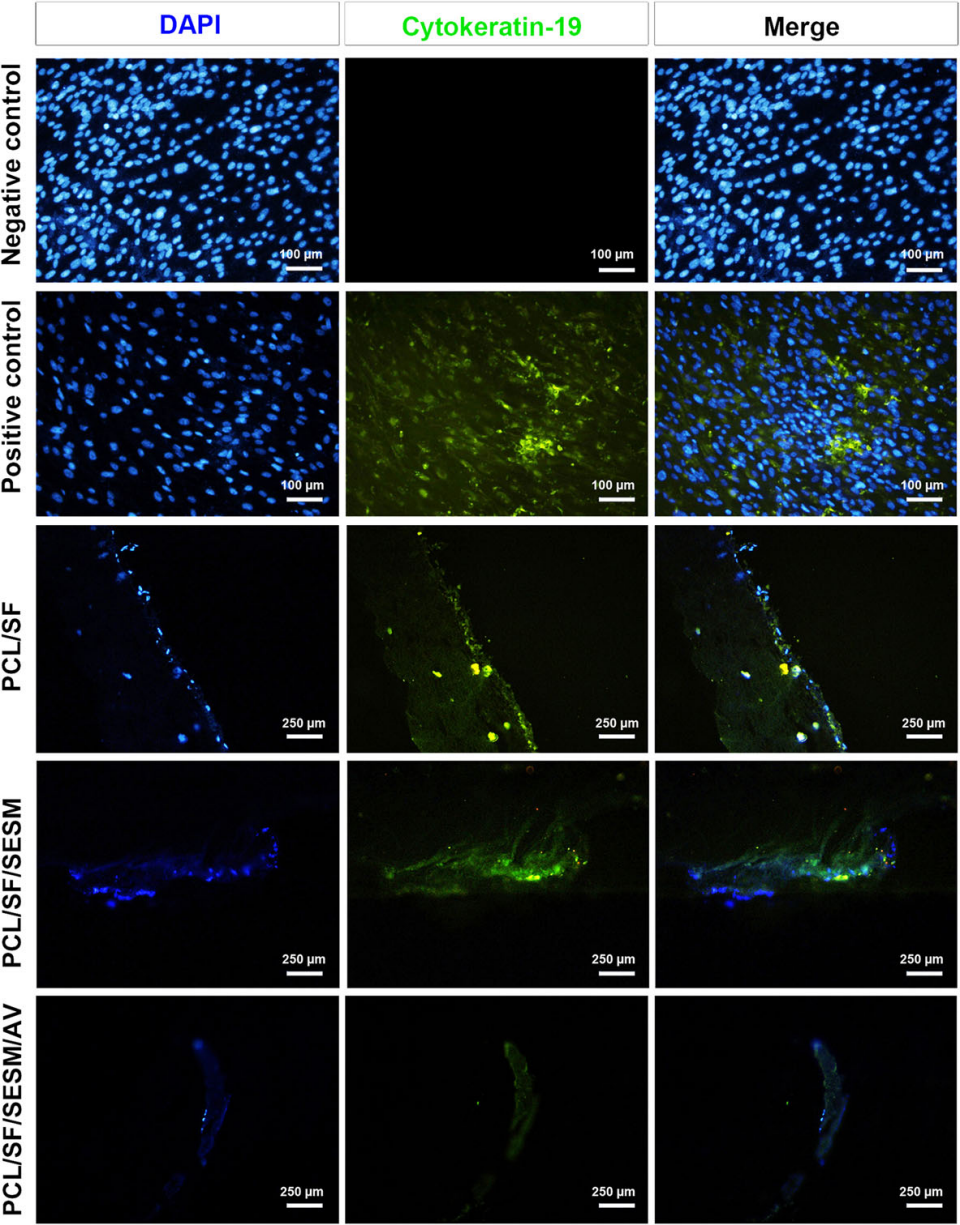
IF imaging of cultured BCCs on different scaffolds based on cytokeratin-19. The culture of BCCs on surfaces PCL/SF and PCL/SF/SESM but not PCL/SF/SESM/AV contributed to the increase of cytokeratin-19 peptide, showing differentiation of cells toward keratinocyte-like cells. This study was performed triplicate (*n* = 3)

IF analysis showed the successful induction of BCCs to keratinocyte-like cells plated on the plastic surface after the addition of the differentiation medium compared to BCCs incubated with growth factor-free medium (Fig. 10). According to our data, we found that the culture of BCCs on scaffolds PCL/SF and PCL/SF/SESM with differentiation medium promoted keratinocyte-like phenotype by the synthesis of cytokeratin-19 peptide, indicated by green-positive cells (Fig. 10). In contrast to these scaffolds, we found lack of cytokeratin-19 in the PCL/SF/SESM/AV group. These data showed that the backbone PCL/SF and its combination with SESM could accelerate the induction of cutaneous progenitor cells toward keratinocyte-like cells.

### Expression of key genes associated with mature skin cells

**Fig. 11:**
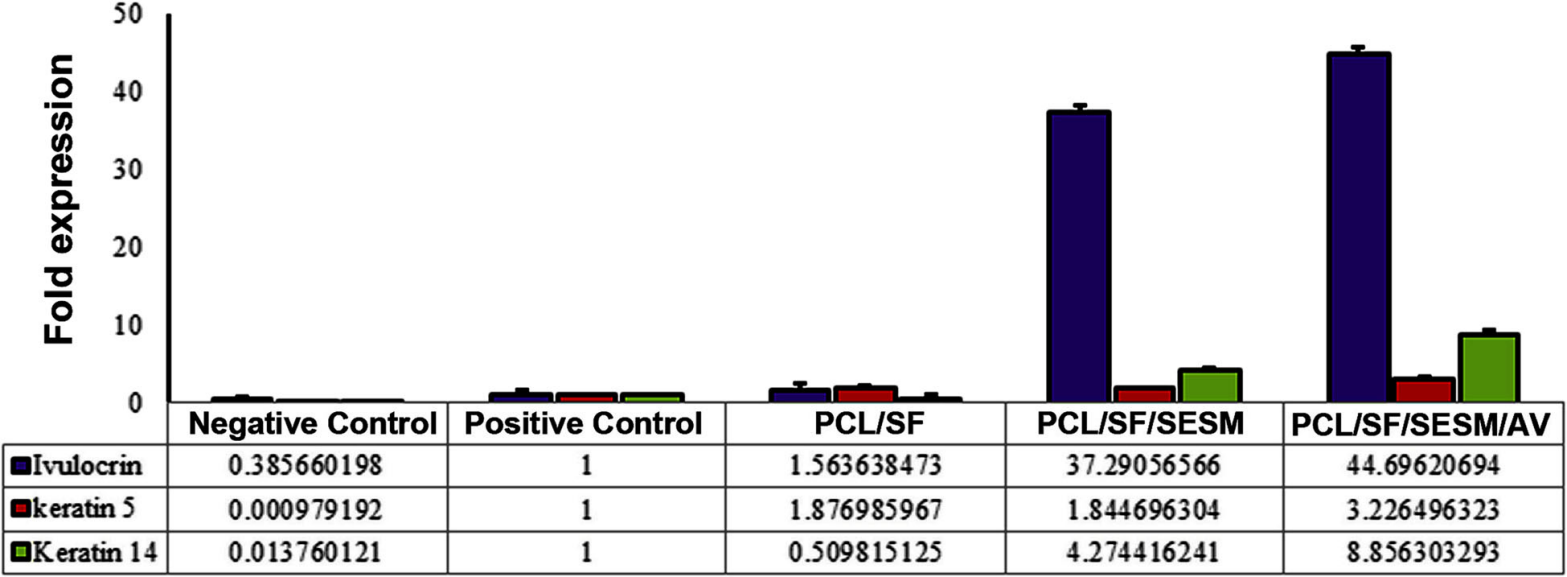
Real-time PCR analysis of BCCs 14 days after plating on different scaffolds showed the superior effects of PCL/SF/SESM and PCL/SF/SESM/AV but not PCL/SF in the induction of involucrin, keratin-14 and -5 transcription required for keratinocyte differentiation. The induction of involucrin was found to be highest compared to the other genes. This study was performed triplicate (*n* = 3)

Real-time PCR analysis showed the potency of all scaffolds to activate the genes associated with the function and phenotype acquisition of keratinocytes (Fig. 11). Based on data, the exposure of cells with differentiation medium induced the transcription of three genes involucrin, keratin-14 and -5 compared to control cells without any factor treatment. The culture of basal cells on scaffolds PCL/SF/SESM/AV and PCL/SF/SESM showed the significant induction of involucrin while these effects were superior in PCL/SF/SESM/AV compared to PCL/SF/SESM and other groups. The stimulatory effect of PCL/SF/SESM/AV on genes keratin-14 and -5more compared to the PCL/SF/SESM and PCL/SF scaffolds (Fig. 11). These features showed that the combination of PCL/SF/SESM/AV had the potential to modulate the activation of genes associated with keratinocyte-like function and activity.

Considering the results from IF and real-time PCR analysis, the culture of basal cells on scaffolds with the mixture of PCL, SF, SESM and AV could induce the protein synthesis and gene activation of the keratinocyte-associated function. However, the pattern of gene expression and protein synthesis is different. Despite the stimulatory effect of PCL/SF/SESM/AV on the induction of genes, the protein level of cytokeratin-19 was not evident compared to other scaffolds. One reason would be related to the consistency of scaffolds during the preparation for IF analysis.

In the current experiment, we did not perform in vivo experiments. We suggest that the ongoing experiments should analyze the potency of these substrates in inducing BCC-to-keratinocyte orientation. This experiment could also be conducted in a prolonged time to investigate the formation of keratinocyte layers.

## Conclusion

The use of semi-synthetic scaffolds based on SESM, SF, and AV could be touted as alternative substrates for the restoration of cutaneous injuries by using BCC. The current experiment highlighted the possible modulatory effects of these scaffold in the promotion of BCC-keratinocyte differentiation. However, prolonged experimental periods are recommended to exactly address the potency of substrates consisted of SESM, SF, and AV in the regeneration of cutaneous pathologies by changing dynamic of BCC toward mature skin cells.

## Supplementary information

**Additional file 1: Figure S1.** SEM image of BCCs single cells on PCL/SF nanofibrous scaffold.

## Abbreviations

AV: *Aloe vera*
BCC: Basal cell carcinoma
DAPI: 4,6-diamidino-2-phenylindole
DMEM-F12: Dulbecco’s modified eagle medium
DMSO: Dimethyl sulfoxide
ECM: Extracellular matrix
ESM: Eggshell membrane
FBS: Fetal bovine serum
FT-IR: Fourier transforms infrared spectroscopy
HFIP: 1, 1, 1, 3, 3, 3-hexafluoro-2-propanol
IF: Immunofluorescence
MTT: 3-(4, 5-dimethylthiazol-2-yl-2, 5-diphenyltetrazolium bromide)
PBS: Phosphate-buffered Saline
PCL: Poly (ɛ-Caprolactone)
SEM: Scanning electron microscopy
SESM: Soluble eggshell membrane
SF: Silk fibroin
TFE: Trifluoroethanol
WCA: Water contact angle
