# Supplementary figures and images for: A novel egg-shell membrane based hybrid nanofibrous scaffold for cutaneous tissue engineering
Source: J Biol Eng. 2019 Oct 26;13:79. doi: 10.1186/s13036-019-0208-x (PMC6815433; doi:10.1186/s13036-019-0208-x)

**
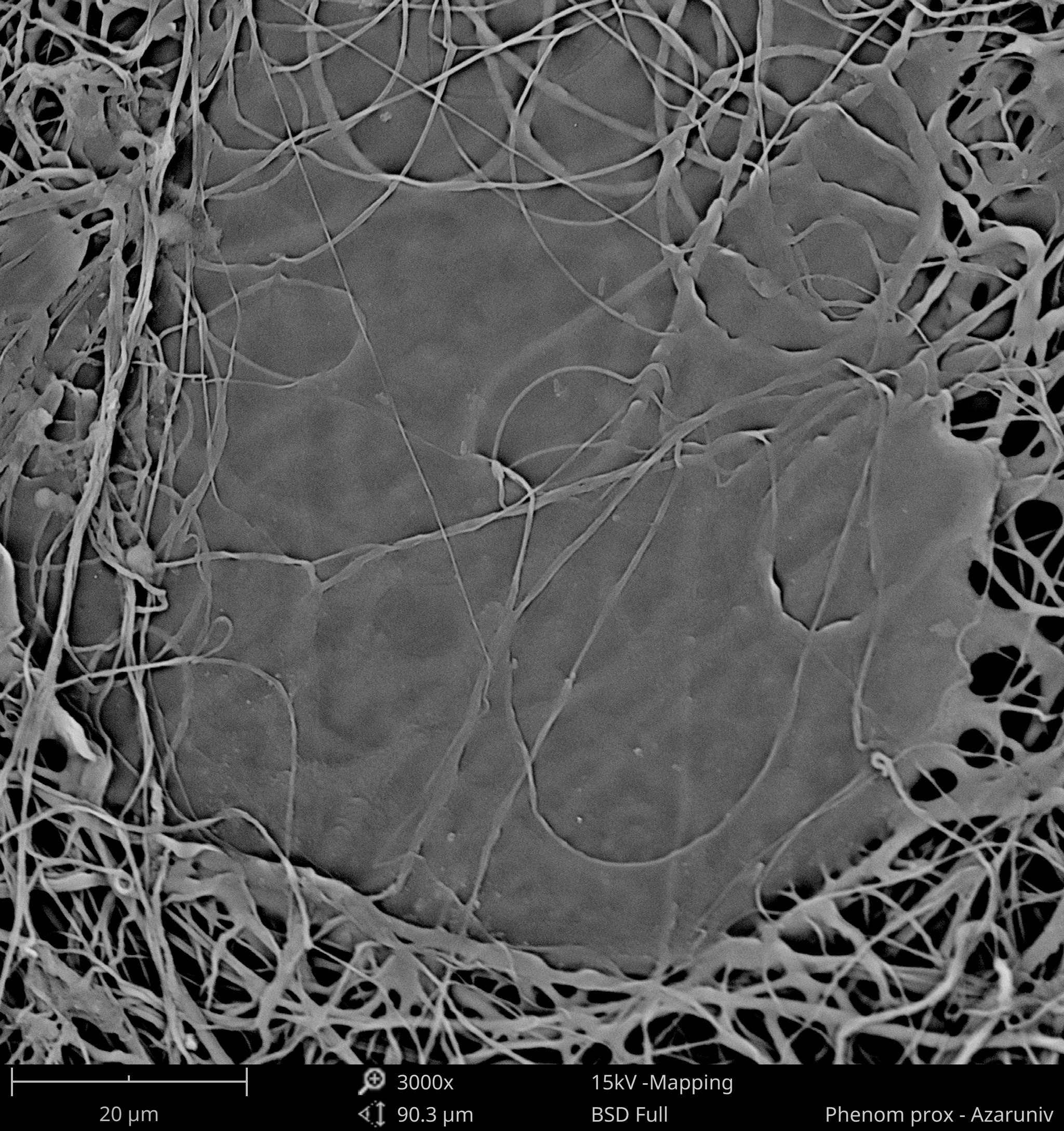


**

Figure S1. SEM image of BCCs single cells on PCL/SF nanofibrous scaffold.

Supplement: Supplementary file 1 — Additional file 1: Figure S1. SEM image of BCCs single cells on PCL/SF nanofibrous scaffold. [file 13036_2019_208_MOESM1_ESM.docx]
